# Impact of dyslipidemic components of metabolic syndrome, adiponectin levels, and anti-diabetes medications on malondialdehyde-modified low-density lipoprotein levels in statin-treated diabetes patients with coronary artery disease

**DOI:** 10.1186/1758-5996-5-77

**Published:** 2013-12-06

**Authors:** Morihiro Matsuda, Ritsu Tamura, Kotaro Kanno, Takatsugu Segawa, Haruyuki Kinoshita, Orie Nishimoto, Hirohiko Nishiyama, Toshiharu Kawamoto

**Affiliations:** 1 Department of Cardiology, National Hospital Organization Kure Medical Center and Chugoku Cancer Center, 3-1 Aoyamacho, Kure, Hiroshima 737-0023, Japan; 2 Department of Internal Medicine, National Hospital Organization Kure Medical Center and Chugoku Cancer Center, 3-1 Aoyamacho, Kure, Hiroshima 737-0023, Japan; 3 Division of Preventive Medicine, Institute for Clinical Research, National Hospital Organization Kure Medical Center and Chugoku Cancer Center, 3-1 Aoyamacho, Kure, Hiroshima 737-0023, Japan

**Keywords:** MDA-LDL, Metabolic syndrome, Triglycerides, HDL cholesterol, Adiponectin, Diabetes mellitus, Coronary artery disease, Statins

## Abstract

**Background:**

A residual risk of cardiovascular disease tends to persist despite standard prevention therapy with statins. This may stem partly from increased oxidized low-density lipoprotein (LDL) levels. However, how oxidized LDL can be further reduced beyond statin therapy in high-risk diabetes patients remains unclear. We aimed to clarify the clinical factors associated with oxidized LDL levels in statin-treated high-risk diabetes patients.

**Methods:**

This cross-sectional observational study included 210 diabetes patients with coronary artery diseases (CAD) who were treated with statins. We determined serum malondialdehyde-modified LDL (MDA-LDL), LDL cholesterol, high-density lipoprotein (HDL) cholesterol, triglyceride (TG), remnant lipoprotein cholesterol, hemoglobin (Hb) A_1c_, adiponectin, and C-reactive protein (CRP) levels and investigated the factors influencing the MDA-LDL level.

**Results:**

In univariate analysis, the MDA-LDL level was significantly correlated with LDL cholesterol (p < 0.0001), TG (p < 0.0001), HDL cholesterol (p = 0.017), and adiponectin (p = 0.001) levels but not with age, body mass index, waist circumference, blood pressure, or HbA_1c_ levels. Even after adjusting for the LDL cholesterol level, the correlations between the MDA-LDL level and the TG, HDL cholesterol, and adiponectin levels were still significant. Among these significant factors, multivariate analysis revealed that the MDA-LDL level was independently associated with the LDL cholesterol, TG, and HDL cholesterol but not with adiponectin levels. The MDA-LDL level was also significantly associated with the CRP level (p = 0.014) and the remnant lipoprotein cholesterol level (p < 0.0001) independently of the LDL cholesterol level. The number of metabolic syndrome (MS) components was significantly associated with the MDA-LDL/LDL cholesterol ratio (p < 0.0001). Furthermore, the use of metformin and α-glucosidase inhibitors was inversely associated with high MDA-LDL levels (p = 0.033 and 0.018, respectively).

**Conclusion:**

In statin-treated diabetes patients with CAD, the MDA-LDL level was significantly correlated with TG and HDL cholesterol levels. Adiponectin level was also significantly associated with the MDA-LDL level, but not independent of the above-mentioned factors. The management of dyslipidemic MS components, including the use of metformin or α-glucosidase inhibitors, may be important for reducing the oxidized LDL levels beyond statin therapy in high-risk diabetes patients.

## Background

Patients with diabetes mellitus (DM) are at high risk for death caused by cardiovascular disease and even higher risk if they have developed coronary artery disease (CAD) [1]. Rather than coronary intervention, prevention therapy is more crucial for reducing the mortality of high-risk patients [2]. According to many clinical trials, it is well established that lowering low-density lipoprotein (LDL) levels with statins is an important prevention strategy for CAD [3,4]. However, even if statins can successfully attenuate the LDL levels, the risk of CAD cannot be eliminated. This residual risk of CAD may involve an increase in the circulating levels of oxidized LDL, which plays an important role in the pathogenesis of atherosclerosis [5,6]. Several studies have demonstrated an association between the prevalence of CAD and the oxidized LDL level [7-10]. Furthermore, the oxidized LDL level may be a useful marker for secondary coronary events in type 2 diabetic patients [11]. Statins reduce the serum oxidized LDL level via LDL-lowering and antioxidative effects [12]. However, in addition to the established prevention therapy with statins, it is unclear how oxidized LDL levels can be further reduced in the high-risk patients with DM.

Patients with metabolic syndrome (MS) are at high risk for CAD and the related mortality [13]. The association between MS and oxidized LDL has recently been demonstrated [14-16]. Adiponectin, a protein released exclusively from adipocytes, has anti-diabetic and anti-atherosclerotic properties [17,18]. Many clinical studies have demonstrated the predictive value of the plasma adiponectin level for severe CAD, myocardial infarction, and mortality [19-21]. In obese individuals, circulating adiponectin levels are decreased [22], which may partly explain the molecular basis of obesity-associated insulin resistance and atherosclerosis. Furthermore, a low adiponectin level is associated with a high triglyceride (TG) level [23], a low high-density lipoprotein (HDL) cholesterol level [24,25], and hypertension [26], suggesting the involvement of adiponectin in the pathogenesis of MS [27]. Systemic oxidative stress levels determined by serum and urinary lipid peroxidation parameters are inversely correlated with serum adiponectin levels in obese people [28]. Moreover, a low serum adiponectin level is associated with a high level of circulating oxidized LDL [29]. However, it remains unclear whether MS and adiponectin levels are still significantly associated with oxidized LDL levels under statin treatment in high-risk DM patients.

Malondialdehyde-modified LDL (MDA-LDL) is recognized as a surrogate marker of oxidized LDL, and it has been suggested that circulating MDA-LDL levels could be a useful indicator for the identification of patients with CAD [7]. In this study, we investigated the clinical factors associated with MDA-LDL levels and evaluated the significance of the MS components and serum adiponectin levels in statin-treated DM patients with CAD. Moreover, we investigated whether any medications affect the MDA-LDL levels in these subjects. Identifying factors that significantly affect the MDA-LDL level should help establish a strategy that can further reduce LDL oxidization in addition to statin therapy, leading to further reduction in the risk of cardiovascular events in high-risk DM patients.

## Methods

### Subjects

We conducted a cross-sectional observational study of 237 DM patients with CAD who underwent coronary angiography or coronary artery computed tomography between 2010 and 2011. All subjects were treated with statins for secondary prevention. We analyzed 210 patients after excluding those who had uncontrolled diabetes with hemoglobin (Hb) A_1c_ > 10.0%, severe hypertriglyceridemia with serum TG > 400 mg/dL, chronic kidney disease with serum creatinine > 2.0 mg/dL, or inflammatory disease with C-reactive protein (CRP) > 3.0 mg/dL. Informed consent was obtained from all subjects. This study protocol was approved by the Ethics Committee of Kure Medical Center.

### Laboratory measurements

Venous blood was drawn from all subjects after an overnight fast. Serum levels of total cholesterol, TG, and HDL cholesterol were determined enzymatically (Sekisui Medical Co., Ltd., Tokyo, Japan). Serum MDA-LDL levels were measured by a sandwich enzyme-linked immunosorbent assay (ELISA) system (Sekisui Medical, Tokyo, Japan) with intra-assay coefficients of variation (CV) of 4.95–7.58% and inter-assay CV of 3.63–7.60%. The HbA_1c_ value was estimated as a National Glycohemoglobin Standardization Program-equivalent percentage calculated using the following formula: HbA_1c_ (%) = 1.02 × HbA_1c_ (Japan Diabetic Society, JDS) (%) + 0.25 [30]. Serum LDL cholesterol levels were calculated using the Friedewald formula. The serum concentration of total adiponectin was measured using a sandwich ELISA system (human adiponectin ELISA kit; Otsuka, Tokushima, Japan). The serum CRP levels were measured using a latex agglutination method (Sekisui Medical, Tokyo, Japan). The serum levels of remnant lipoprotein cholesterol were determined using a homogenous assay (RemL-C assay; Kyowa Medex, Tokyo, Japan) [31].

### Definition of DM and MS

DM patients were defined according to the Japanese criteria [30] and/or prior use of anti-diabetic medication. The components of MS other than high plasma fasting glucose (all patients had DM) were defined according to the Japanese criteria [32] as follows: (1) waist circumference ≥ 85 cm for men or ≥ 90 cm for women; (2) elevated serum TG ≥ 150 mg/dL; (3) reduced serum HDL cholesterol < 40 mg/dL; and (4) elevated systolic blood pressure ≥ 130 mmHg and/or diastolic blood pressure ≥ 85 mmHg and/or prior use of antihypertensive medication.

### Statistical analyses

Data are expressed as mean ± standard deviation (SD). Single and multiple regression analyses were performed to evaluate the association between MDA-LDL levels and the indicated variables. Analysis of variance (ANOVA) with Tukey-Kramer’s honestly significant difference test was used to evaluate the association between the number of MS components and the MDA-LDL/LDL cholesterol ratios. A multivariable logistic model was used to assess the association between a high MDA-LDL level and the indicated medications. Statistical analyses were performed using JMP for Windows (version 9; SAS Institute, Cary, NC). Statistical significance was defined by a p value of <0.05.

## Results

### Subject characteristics

The characteristics of the subjects and the medications prescribed are shown in Table 1. Because all subjects were treated with statins, the average LDL cholesterol was as low as 92.6 mg/dL. The statins used were atorvastatin 5–20 mg (37% of patients), pravastatin 5–20 mg (27%), rosuvastatin 2.5–10 mg (26%), pitavastatin 2–3 mg (7%), simvastatin 5–10 mg (2%), and fluvastatin 20 mg (1%). Other anti-hyperlipidemic agents were prescribed in a small number of patients: ezetimibe for 1 patient and eicosapentaenoic acid (EPA) for 9 patients. The mean HbA_1c_ level was 6.9%.

**Table 1:** Subject characteristics

**N**	**210**
Age ─ years	70.6 ± 8.7
Male sex ─ no. (%)	136 (64.7)
Morphometric findings	
Body mass index ─ kg/m^2^	24.6 ± 4.1
Waist circumference ─ cm	88.6 ± 10.5
Blood pressure	
Systolic ─ mmHg	133.5 ± 19.6
Diastolic ─ mmHg	73.9 ± 13.5
Laboratory measurements	
Serum creatinine ─ mg/dL	0.9 ± 0.2
Total cholesterol ─ mg/dL	166.4 ± 31.7
Triglycerides ─ mg/dL	
Median (interquartile range)	105 (77–143)
HDL cholesterol ─ mg/dL	50.1 ± 13.7
LDL cholesterol ─ mg/dL	92.6 ± 23.0
MDA-LDL ─ U/L	99.0 ± 39.0
Hemoglobin A_1c_ ─ %	6.9 ± 1.0
Adiponectin ─ μg/dL	
Median (interquartile range)	13.3 (8.5 − 24.1)
Current medications ─ no. (%)	
Statins	210 (100)
ACE-Is/ARBs	152 (72.4)
β-Blockers	132 (62.9)
Ca channel blockers	85 (40.5)
Metformin	84 (40.0)
Pioglitazone	79 (37.6)
α-Glucosidase inhibitors	70 (33.3)
Sulfonylurea	61 (29.0)
Insulin therapy	37 (17.6)
Phenylalanine derivatives	14 (6.7)
DPP4 inhibitors	15 (7.1)
EPA	9 (4.3)
Ezetimibe	1 (0.5)

Values represent means ± SD unless indicated otherwise.

*Abbreviations:ARB* angiotensin II receptor blocker, *ACE-I* angiotensin-converting enzyme inhibitor, *DPP4* dipeptidyl peptidase-4; *EPA* eicosapentaenoic acid, *HDL* high-density lipoprotein, *LDL* low-density lipoprotein, *MDA-LDL* malondialdehyde-modified LDL.

### Associations between the MDA-LDL level and various risk factors

**Figure 1:**
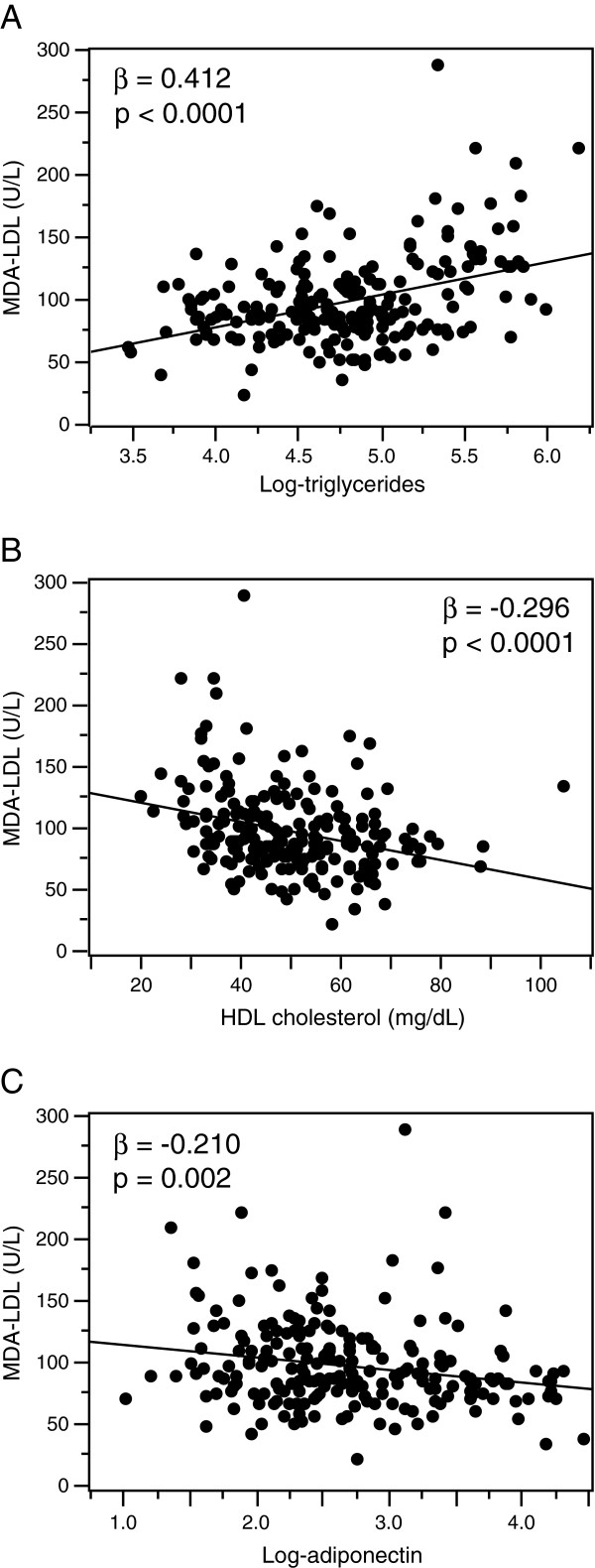
**Correlations between the malondialdehyde-modified low-density lipoprotein (MDA-LDL) level and associated factors after adjusting for LDL cholesterol levels.** The MDA-LDL level (U/L) was significantly correlated with the levels of log-transformed triglycerides **(A)**, high-density lipoprotein (HDL) cholesterol (mg/dL) **(B)**, and log-transformed adiponectin **(C)** with the indicated partial correlation coefficient (β) and p value. Statistical analyses were performed by multiple regression models adjusted for LDL cholesterol level.

The MDA-LDL level was significantly correlated with the levels of LDL cholesterol, TG, HDL cholesterol, and adiponectin (p < 0.0001, p < 0.0001, p = 0.017, and p = 0.001, respectively) but not with age, body mass index (BMI), waist circumference, blood pressure, or creatinine or HbA_1c_ levels in single regression analysis (Table 2). Even after adjusting for the LDL cholesterol level, the MDA-LDL level was significantly correlated with the levels of TG, HDL cholesterol, and adiponectin (p < 0.0001, p < 0.0001, and p = 0.002, respectively) (Figure 1). Multiple regression analysis revealed that the levels of LDL cholesterol, HDL cholesterol, and TG were independently associated with the MDA-LDL level (Table 3, model 1). The adiponectin level, however, was not significantly associated with the MDA-LDL level in this analysis (Table 3, model 1).

**Table 2:** Relationship between the level of MDA-LDL and various parameters in univariate analyses

	**r**	**p**
Age	0.010	0.887
Body mass index	−0.023	0.739
Waist circumference	0.025	0.724
Systolic blood pressure	0.075	0.281
Creatinine	0.036	0.600
Hemoglobin A1c	0.068	0.330
LDL cholesterol	0.458	<0.0001
Triglycerides*	0.435	<0.0001
HDL cholesterol	−0.164	0.017
Adiponectin*	−0.227	0.001

*Abbreviations:HDL* high-density lipoprotein, *LDL* low-density lipoprotein, *MDA-LDL* malondialdehyde-modified LDL.

Statistical analyses were performed using simple regression.

*Log-transformed values were subjected to statistical analyses.

**Table 3:** Impact of LDL cholesterol, triglycerides, HDL cholesterol, and adiponectin on the level of MDA-LDL in the multivariate analyses

	**Model 1**		**Model 2**	
	**β**	**p**	**β**	**p**
LDL cholesterol	0.452	<0.0001	0.444	<0.0001
Triglycerides*	0.317	<0.0001	0.318	<0.0001
HDL cholesterol	−0.137	0.049	−0.139	0.048
Adiponectin*	−0.049	0.481	−0.032	0.648

*Abbreviations:HDL* high-density lipoprotein, *LDL* low-density lipoprotein, *MDA-LDL* malondialdehyde-modified LDL.

In model 1 (R^2^ = 0.361), LDL cholesterol, triglycerides, HDL cholesterol, and adiponectin were included as the variables in the multiple regression analysis. In model 2 (R^2^ = 0.365), medications (metformin and α-glucosidase inhibitors) were added to the variables for the model 1 analysis.

*Log-transformed values were subjected to statistical analyses.

**Figure 2:**
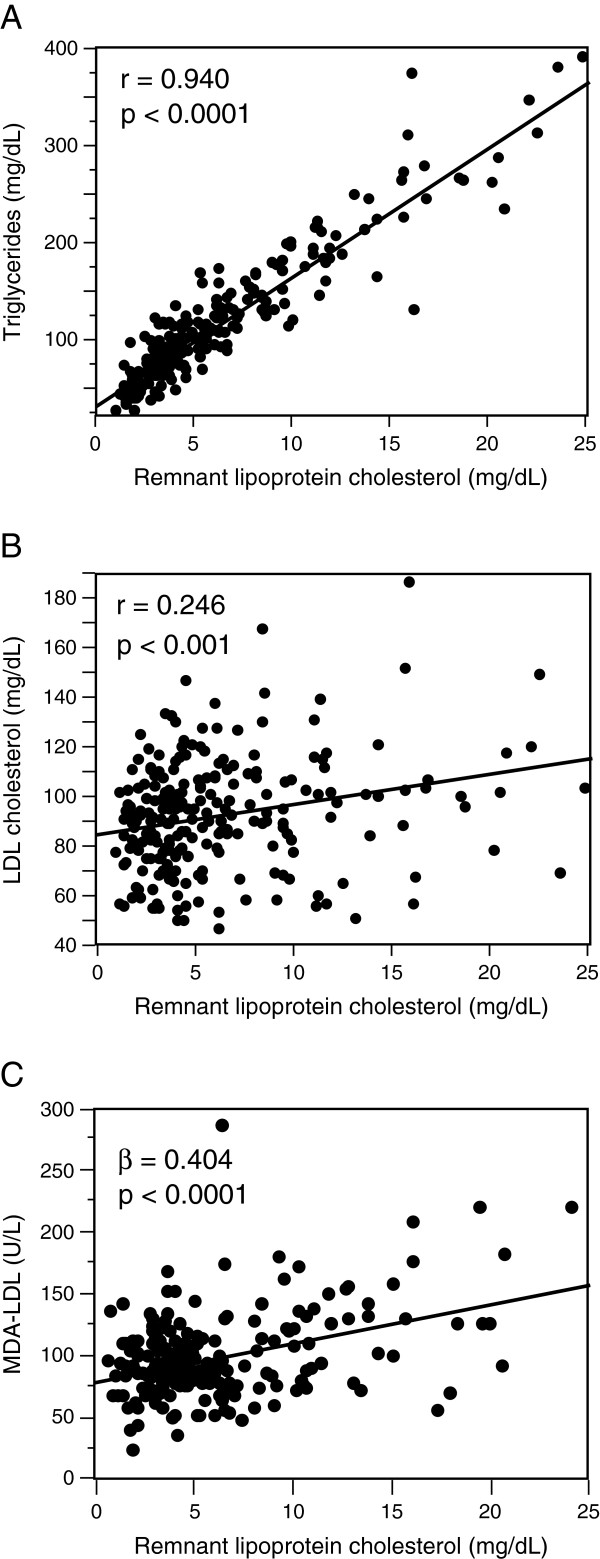
**Associations of the remnant lipoprotein cholesterol level with the levels of triglycerides, low-density lipoprotein (LDL) cholesterol, and malondialdehyde-modified LDL (MDA-LDL). A** and **B**, The remnant lipoprotein cholesterol level (mg/dL) was significantly correlated with the levels of triglycerides (mg/dL) **(A)** and LDL cholesterol (mg/dL) **(B)** with the indicated correlation coefficient (r) and p value in simple regression analyses. **C**, The remnant lipoprotein cholesterol level (mg/dL) was significantly correlated with the MDA-LDL level (U/L) with the indicated partial correlation coefficient (β) and p value in a multiple regression model adjusted for LDL cholesterol level.

To understand in more detail the basis of the association between the MDA-LDL and TG levels, we investigated the level of remnant lipoprotein cholesterol. The remnant lipoprotein cholesterol level was significantly correlated with the TG level with a very high correlation coefficient (r = 0.940) and the LDL cholesterol level with a relatively low correlation coefficient (r = 0.246) (Figure 2A and B), suggesting that the remnant lipoproteins could be the major lipoproteins carrying TG in the fasting state upon statin treatment. Furthermore, the remnant lipoprotein cholesterol level was significantly correlated with the MDA-LDL level independently of the LDL cholesterol level in a multiple regression model (Figure 2C).

### Association between the MDA-LDL/LDL cholesterol ratio and the number of MS components

**Figure 3:**
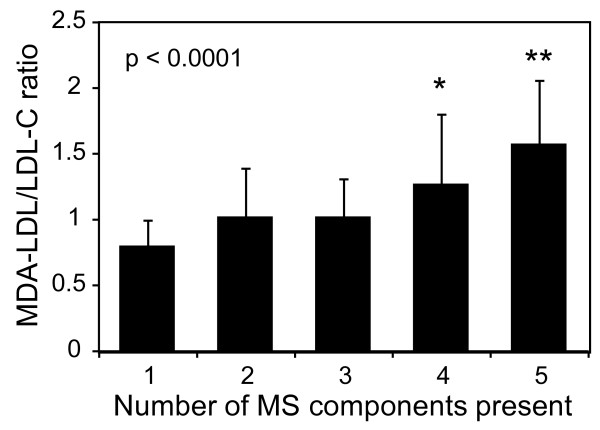
**Association between the malondialdehyde-modified low-density lipoprotein (MDA-LDL)/LDL cholesterol (C) ratio and the number of metabolic syndrome (MS) components.** The MDA-LDL/LDL-C ratios were compared in patients with the indicated number of MS components. Data are expressed as means ± SD. Statistical analyses were performed by analysis of variance with Tukey-Kramer’s honestly significant difference test. *, p < 0.05 vs. 1, 2, or 3; **, p < 0.001 vs. 1, 2, or 3.

The number of MS components present was significantly associated with the MDA-LDL/LDL cholesterol ratio (p < 0.0001) (Figure 3). Patients with ≥4 of the 5 MS component factors had a significantly higher MDA-LDL/LDL cholesterol ratio than those with fewer factors (p < 0.05 for 4 vs. 1, 2, or 3 MS components; p < 0.001 for 5 vs. 1, 2, or 3 MS components).

### Association between a high MDA-LDL level and inflammation

Because inflammation plays a pivotal role in the pathogenesis of both MS and atherosclerosis, we investigated the association between CRP and MDA-LDL levels. A high CRP level of ≥ 0.2 mg/dL was significantly associated with a high MDA-LDL level (defined by a median value of > 91 U/L), compared with a low CRP level of < 0.1 mg/dL (Table 4). This association remained significant even after adjusting for the LDL cholesterol level (Table 4). However, this significance was diminished by adjusting for both LDL cholesterol and TG levels and was eliminated after adjusting for LDL cholesterol, TG, and HDL cholesterol levels (Table 4).

**Table 4:** Association between CRP levels and high MDA-LDL level in univariate and multivariate logistic analyses

	**CRP concentration, mg/dL**			
	**<0.1**	**0.1–0.2**	**≥0.2**	p*
Univariate, OR (95% CI)	1	1.77 (0.89–3.55)	3.40 (1.54–8.02)	0.006
Multivariate, OR (95% CI)				
Adjusted for LDL-C	1	1.43 (0.68–3.01)	3.44 (1.49–8.45)	0.014
Adjusted for LDL-C, TG	1	1.08 (0.49–2.35)	2.74 (1.14–6.95)	0.071
Adjusted for LDL-C, TG, HDL-C	1	0.95 (0.42–2.09)	2.41 (0.98–6.20)	0.124

*Abbreviations:CI* confidence interval, *CRP* C-reactive protein, *HDL-C* high-density lipoprotein cholesterol, *LDL-C* low-density lipoprotein cholesterol, *MDA-LDL* malondialdehyde-modified LDL, *OR* odds ratio; *TG* triglycerides.

Univariate and multivariate logistic analyses with an adjustment for the indicated parameters were performed. A high MDA-LDL level was defined by a median value of >91 U/L.

*Likelihood ratio test for trend.

### Association between a high MDA-LDL level and medications combined with statins

In a multivariate logistic analysis adjusted for LDL cholesterol, TG, HDL cholesterol, and adiponectin levels, we found, among the medications used in combination with statins, that the use of metformin and α-glucosidase inhibitors (α-GIs) was inversely associated with a high MDA-LDL level (defined by a median value of > 91 U/L) (Table 5). Conversely, the associations of LDL cholesterol, TG, and HDL cholesterol with MDA-LDL remained significant in another multivariate model even after adjustment for the use of metformin and α-GIs (Table 3, model 2).

**Table 5:** Multivariate logistic analysis of the relationship between medications used with statins and high MDA-LDL level

	**Adjusted OR (95% CI)**	**p**
ACE-Is or ARBs	1.29 (0.61–2.78)	0.508
β-Blockers	1.04 (0.51–2.12)	0.923
Ca channel blockers	1.23 (0.62–2.45)	0.548
Metformin	0.48 (0.24–0.94)	0.032
Pioglitazone	0.61 (0.28–1.28)	0.190
α-Glucosidase inhibitors	0.43 (0.21–0.87)	0.018
Sulfonylurea	1.65 (0.76–3.66)	0.205
Insulin therapy	0.51 (0.21–1.20)	0.124
Other variables		
LDL cholesterol	241.6 (22.0–3289.8)*	<0.0001
Triglycerides	12.95 (2.02–93.09)*	0.007
HDL cholesterol	0.10 (0.00–0.98)*	0.048
Adiponectin	1.18 (0.19–7.12)*	0.859

*Abbreviations:ACE-I* angiotensin-converting enzyme inhibitor, *ARB* angiotensin II receptor blocker, *CI* confidence interval; *HDL* high-density lipoprotein, *LDL* low-density lipoprotein, *MDA-LDL* malondialdehyde-modified LDL, *OR* odds ratio.

Multivariate logistic regression analysis was performed with an adjustment for all indicated medicines and the levels of LDL cholesterol, triglycerides, HDL cholesterol, and adiponectin. A high MDA-LDL level was defined by a median value of >91 U/L.

*Range OR (95% CI).

## Discussion

In this study, the level of MDA-LDL was significantly correlated with the levels of TG, HDL cholesterol, and adiponectin, independent of the LDL cholesterol level, in statin-treated DM patients with CAD. However, there was no significant association between the level of adiponectin and the level of MDA-LDL when the regression was adjusted for the levels of LDL cholesterol, TG, and HDL cholesterol. An increasing number of MS components resulted in a higher MDA-LDL/LDL cholesterol ratio. Among the medications used in combination with statins, the use of metformin and α-GIs was inversely associated with a high MDA-LDL level in multivariate logistic analysis.

This study revealed that TG and HDL cholesterol were significantly correlated with MDA-LDL levels independent of the LDL cholesterol level in statin-treated high-risk DM patients. An increased TG level and decreased HDL cholesterol level are observed due to insulin resistance, and hence, they are recognized as dyslipidemic components of MS. The prolonged presence of increased TG-rich lipoproteins in the circulation induces oxidative stress in the endothelium [33,34]. The current study revealed the significant association between the levels of MDA-LDL and remnant lipoprotein cholesterol. Activation of lectin-like oxidized LDL receptor-1 by remnant lipoprotein particles induces nicotinamide adenine dinucleotide phosphate (NADPH) oxidase-dependent production of superoxide in endothelial cells [35], which may explain the significant association between LDL oxidation and remnant lipoproteins rich in TG. Moreover, HDL protects against the oxidation of LDL [36]. Taken together, these results might explain the close association between oxidized LDL and the dyslipidemic components of MS. Holvoet et al. demonstrated the association between various features of MS and oxidized LDL in well-functioning elderly people [14]. Our study, however, indicated that in statin-treated DM patients, the level of MDA-LDL was associated with the dyslipidemic components of MS but not with other features of MS, including blood pressure, waist circumference, or the level of HbA_1c_ (instead of fasting glucose). Nevertheless, patients with ≥4 of the 5 MS factors had a significantly higher MDA-LDL/LDL cholesterol ratio than those with fewer factors. This suggests that the additional presence of hypertension and/or abdominal obesity can somehow aggravate the oxidation of LDL. These findings also suggest that accumulation of these MS-constituting factors should be attenuated to reduce the oxidation of LDL even with statin treatment in high-risk DM patients.

Inflammation plays a pivotal role in the progression of atherosclerosis. CRP ≥ 0.2 mg/dL is significantly associated with the incidence of cardiovascular events [37,38]. The current data indicated that a high CRP level of ≥0.2 mg/dL was significantly associated with a high level of MDA-LDL independently of LDL cholesterol. On the other hand, additional adjustment for TG and HDL cholesterol levels eliminated the significance of the association between CRP and MDA-LDL levels, suggesting that the inflammation may affect LDL oxidation, possibly through the dyslipidemic changes of MS.

We previously demonstrated that oxidative stress was closely associated with a low adiponectin level [28]. Adiponectin production from adipocytes was suppressed by reactive oxygen species but reversed by the addition of an antioxidant agent [28]. Treating KKAy obese mice with apocynin, an NADPH oxidase inhibitor, prevented the reduction in adiponectin level by suppressing oxidative stress, leading to an improvement in insulin resistance and dyslipidemia [28]. Moreover, adiponectin protected against damage induced by oxidative stress in the vascular walls and myocardium [39,40]. Thus, adiponectin is likely to be closely linked to oxidative stress. A low adiponectin level was inversely associated with the oxidized LDL level [29]. Similarly, in our study, the level of adiponectin was significantly correlated with the MDA-LDL level in the regression analysis adjusted for the LDL cholesterol level. However, this correlation was not significant after we adjusted for the levels of TG and HDL cholesterol, possibly because the level of adiponectin is negatively correlated with the TG level and positively correlated with the HDL cholesterol level [27]. Collectively, the relationship between the adiponectin and oxidized LDL levels may be influenced by the TG and HDL cholesterol levels.

Keaney et al. demonstrated that obesity was associated with systemic oxidative stress as assessed by the level of urinary 8-epi-prostaglandin F2α (PGF2α) [41]. Similarly, we demonstrated that the level of serum thiobarbituric acid reactive substance and urinary 8-epi-PGF2α were positively correlated with BMI independent of diabetes in humans [28]. However, in the present study, the MDA-LDL level was not significantly associated with BMI or waist circumference despite a significant association with obesity-related factors such as TG, HDL cholesterol, and adiponectin levels. Both MDA-LDL and lipid peroxidation parameters indicate the level of oxidative stress. However, they may have different associations with clinical parameters such as LDL cholesterol level or BMI because of their different origins.

In the present study, the use of metformin was inversely associated with a high MDA-LDL level, independent of LDL cholesterol, TG, and HDL cholesterol levels. Metformin reduces the synthesis of hepatic very-low-density lipoprotein and the production of glucose, which is mediated by 5’-adenosine monophosphate-activated protein kinase [42], and leads to increased insulin sensitivity and reduction in the plasma TG levels. This might partly contribute to the reduction in MDA-LDL levels, although the precise mechanism remains to be clarified. Similarly, the use of α-GIs was inversely associated with a high MDA-LDL level. The α-GIs reportedly suppress oxidative stress in the vascular wall by suppressing postprandial hyperglycemia [33,43], which might explain the reduction of MDA-LDL. Despite increasing the circulating levels of adiponectin [44,45] as well as several antioxidative enzymes [46-48], the use of pioglitazone was not significantly associated with the MDA-LDL level in this analysis. The antioxidative actions of pioglitazone might not be as effective as metformin or α-GIs in suppressing LDL oxidation. Further investigation of this issue is required. Among various anti-diabetic agents, metformin and α-GIs have been proven to be effective in the primary prevention of cardiovascular disease in large-scale clinical trials [49,50]. Importantly, they suppress weight gain [49,50]. An improvement in insulin resistance and postprandial hyperglycemia with suppression of weight gain may be important for reducing LDL oxidation, which should contribute to the prevention of cardiovascular events.

When the 9 patients who had taken EPA were excluded from the univariate analysis, the correlations of the LDL cholesterol, TG, HDL cholesterol, and adiponectin levels with the MDA-LDL level remained significant. However, in the multivariate analysis, the association of HDL cholesterol with MDA-LDL was weakened from p = 0.049 to p = 0.065, suggesting that EPA might affect the MDA-LDL level, possibly through an influence on TG and HDL cholesterol levels. Therefore, further analysis in a large population is required to resolve this issue.

This study had several limitations. The subjects in this study were relatively elderly patients (mean age, 70.6 years), and any differences with respect to age are unknown. In addition, this was a cross-sectional observational study conducted in a single center. Multicenter prospective cohort studies and randomized controlled trials are essential to determine the clinical determinants of oxidized LDL and effective medications for reducing LDL oxidation.

## Conclusion

DM patients with CAD are at very high risk for cardiovascular events. Hence, they require a risk-reducing strategy beyond the standard prevention therapy with statins. In these patients treated with statins, the circulating MDA-LDL levels were significantly correlated with the TG and HDL cholesterol levels. Adiponectin levels were also significantly correlated with MDA-LDL levels, but not independent of TG and HDL cholesterol. Furthermore, treatment with metformin or α-GIs might be associated with a reduction in MDA-LDL levels. Collectively, this study suggests that in addition to statin therapy, the management of dyslipidemic MS components, including use of metformin or α-GIs, is important for reducing the oxidization of LDL and, ultimately, the risk of cardiovascular events in high-risk DM patients.

## Abbreviations

ANOVA: Analysis of variance; BMI: Body mass index; CAD: Coronary artery disease; CRP: C-reactive protein; DM: Diabetes mellitus; 8-epi-PGF2α: 8-epi-prostaglandin F2α; Hb: Hemoglobin; HDL: High-density lipoprotein; LDL: Low-density lipoprotein; MDA-LDL: Malondialdehyde-modified LDL; MS: Metabolic syndrome; NADPH: Nicotinamide adenine dinucleotide phosphate; TG: Triglyceride.

## Competing interests

The authors declare that they have no competing interests.

## Authors’ contributions

MM, RT, and TK have made substantial contributions to the conception and design. KK, TS, HK, ON, and HN have made substantial contributions to data acquisition. MM, RT, and TK have contributed to the analysis and interpretation of data. MM was involved in drafting the manuscript. RT, KK, TS, HK, ON, HN, and TK provided critical revision for important intellectual content. All authors have given final approval for publishing.
